# Evaluating Status Change of Soil Potassium from Path Model

**DOI:** 10.1371/journal.pone.0076712

**Published:** 2013-10-30

**Authors:** Wenming He, Fang Chen

**Affiliations:** 1 Key Laboratory of Aquatic Botany and Watershed Ecology, Wuhan Botanical Garden, Chinese Academy of Sciences, Moshan, Wuchang, Wuhan, Hubei Province, China; 2 Graduate University of Chinese Academy of Sciences, Beijing, China; 3 International Plant Nutrition Institute, Wuhan, China; Institute for Plant Protection (IPP), CNR, Italy

## Abstract

The purpose of this study is to determine critical environmental parameters of soil K availability and to quantify those contributors by using a proposed path model. In this study, plot experiments were designed into different treatments, and soil samples were collected and further analyzed in laboratory to investigate soil properties influence on soil potassium forms (water soluble K, exchangeable K, non-exchangeable K). Furthermore, path analysis based on proposed path model was carried out to evaluate the relationship between potassium forms and soil properties. Research findings were achieved as followings. Firstly, key direct factors were soil S, ratio of sodium-potassium (Na/K), the chemical index of alteration (CIA), Soil Organic Matter in soil solution (SOM), Na and total nitrogen in soil solution (TN), and key indirect factors were Carbonate (CO_3_), Mg, pH, Na, S, and SOM. Secondly, path model can effectively determine direction and quantities of potassium status changes between Exchangeable potassium (eK), Non-exchangeable potassium (neK) and water-soluble potassium (wsK) under influences of specific environmental parameters. In reversible equilibrium state of 
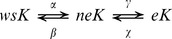
, K balance state was inclined to be moved into β and χ directions in treatments of potassium shortage. However in reversible equilibrium of 
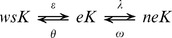
, K balance state was inclined to be moved into θ and λ directions in treatments of water shortage. Results showed that the proposed path model was able to quantitatively disclose moving direction of K status and quantify its equilibrium threshold. It provided a theoretical and practical basis for scientific and effective fertilization in agricultural plants growth.

## Introduction

The status and transformation of potassium in the soil is of great significance to crop growth [1], [2]. Investigations on potassium status gained a numerous achievements[3], [4]. The effectiveness of potassium in soil is controlled by four forms, e.g., mineral potassium, non-exchangeable potassium (neK), exchangeable potassium (eK) and water-soluble potassium (wsK) which can be transformed into each other [5]–[8]. Absorption capacity of crops and application of farming fertilizer have influence on the potassium forms, thus impact on the release and fixation of potassium in the soil [9]. Within these different forms of soil potassium, there exists a complex and dynamic chemical balance, and it greatly depends on the situation of each form and its environment conditions [10]–[13]. However, there are few experimental studies on ternary systems involving K^+^ in spite of the plant nutritional importance of K^+^ in soils [3], [5], [14]. Research indicated that the amount of K ions retained on the solid phase of soil was much larger than that dissolved in the soil solution. It suggested that a root segment become easily permeable to Ca^2+^ followed by Na^+^ and K^+^ with aging [15], [16]. Thus, it is indispensable to take into account the cation exchange processes in modeling the response of the soil solution to fertilizer application and transport of cationic solutes in soils.

The transform of soil non-exchangeable potassium to soil exchangeable potassium and soil soluble potassium is a slowly process. It is hard to be determined by using the routine method of ion exchange, although it shows that sodium tetraphenylborate method is more accurate than conventional methods to reflect changes in soil potassium through long-term field fixed experiments of potash fertilization [17], [18]. Path analysis is a statistical technique that distinguishes coefficient and causation by partitioning coefficients into direct and indirect effects. Researchers used path analysis to analysis phosphorus retention capacity in allophanic and non-allophanic andisols and soil organic matter effects on phosphorus sorption is successful [19], [20]. The method of path coefficients, which was proposed by Wright (1921), was effective in disclosing relationships between variables by diagrams [21]–[25]. However, path coefficients approach has not yet applied to the study on soil potassium. The potassium absorb efficiency of plant root is affected by multiple factors [26], so it is not suitable to make estimation by use a single parameter [27]–[29]. The above mentioned studies focused on qualitatively descriptive analysis of single factor or multifactor, and failed to quantitatively disclose the dynamic process of potassium status [30].

In this study, we conducted soil K ions exchange experiments for soil samples and further calculated their selectivity coefficients, and explored potassium status on basis of path model to determine dynamic changes of potassium status and specific environmental parameters. The model assumptions were related to the adsorption mechanism at molecular level. The first step, we attempted to examine different indicators of potassium in the soils, and then to analyze direct and indirect correlations between water-soluble potassium (wsK), non-exchangeable potassium (neK) and exchangeable potassium (eK), and further to calculate path coefficients of different potassium status, finally to construct path model for describing the changes of potassium status. The model was calibrated and validated by using 48 cotton rhizosphere/nonrhizosphere soils samples to test its accuracy. We quantitatively investigated on interaction process of plant-soil-microorganisms and relationship between environmental parameters and potassium release through path coefficient model, and the model was used to predict the multi-component ion exchange equilibrium in soil.

## Materials and Methods

Hereby, I, along with coauthor, confirm that no specific permissions were required for our experiment locations/activities since this experiment field belongs to our institute and for scientific research only. And the field studies did not involve endangered or protected species.

In this study, meadow soil was selected as experimental agents in order to investigate potassium nutrient supply and physiological mechanism (Table 1). Rhizosphere soil samples were collected from 90 days cotton plant. When collecting soil samples, we firstly loosed root zone soil to collect rhizosphere soil, and uprooted whole plant cotton, and then gently shook off root zone soil, and finally get root surface adhesion soil. The non-rhizosphere soil samples were collected 10–15 cm depth from surface.

**Table 1:** Properties of mead soil for experiments.

Soil properties		Average value
**Mean compact density (g·cm^−3^)**		1.54
**Particle composition**	**Clay (<0.002 mm, %)**	16.8
	**Sand (2-0.05 mm, %)**	61.7
	**Silt (0.05-0.002 mm, %)**	21.4
**Mineral composition**	**Smectite (%)**	4.0
	**Vermienlite (%)**	25.0
	**Intergrade mineral hydromica (1.4 nm, %)**	49.0
	**Kaolinite (%)**	19.0
**Cations composition**	**exchangeable Ca (mmol·kg^−1^)**	35.4
	**exchangeable Mg (mmol·kg^−1^)**	18.1
	**exchangeable Na (mmol·kg^−1^)**	2.0
	**FeO (noncrystalline iron extracted with Tamm's solution, g·kg^−1^)**	49.0

**Figure 1:**
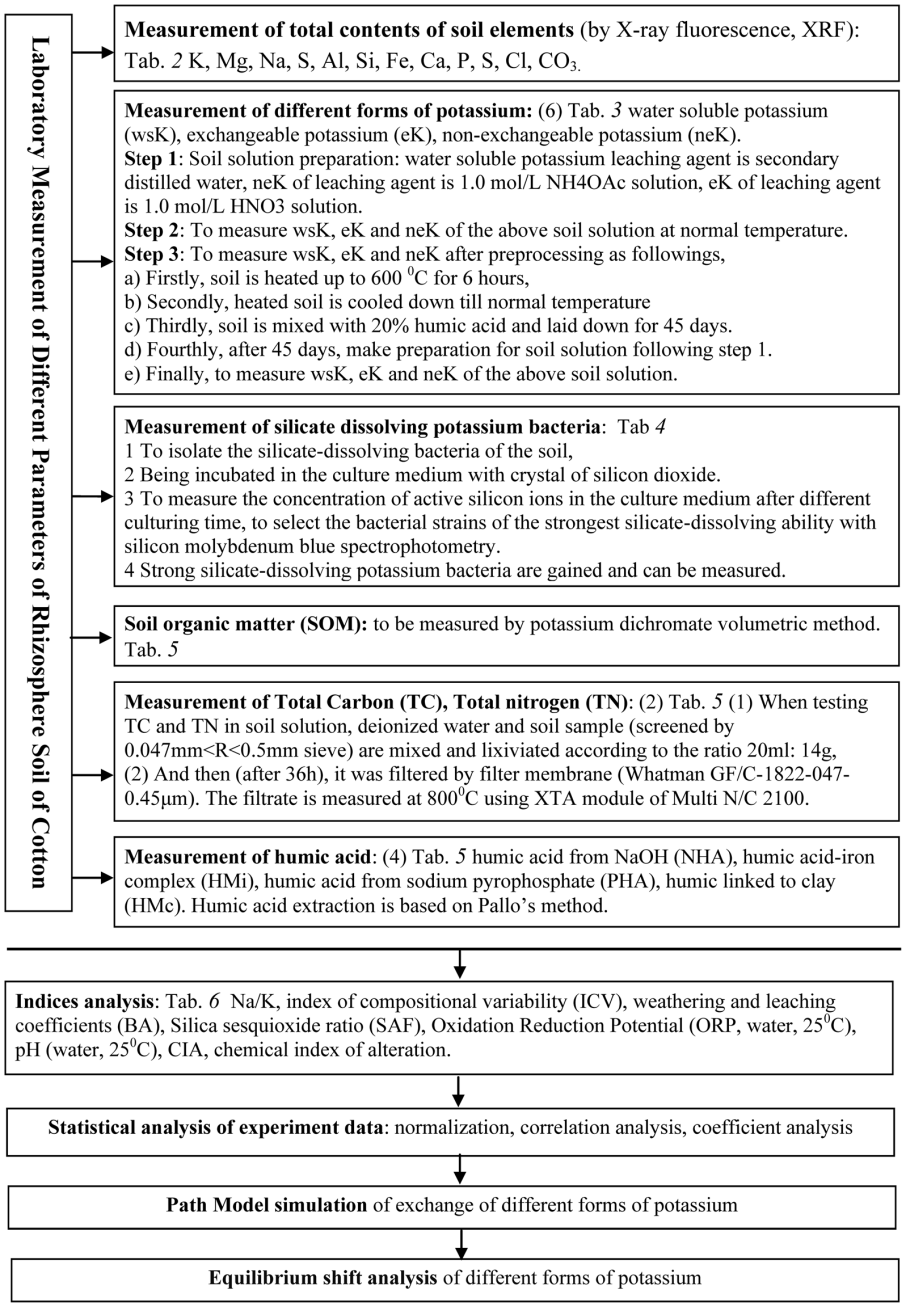
Workflow of evaluating status changes of soil potassium from Path Model.

**Figure 2:**
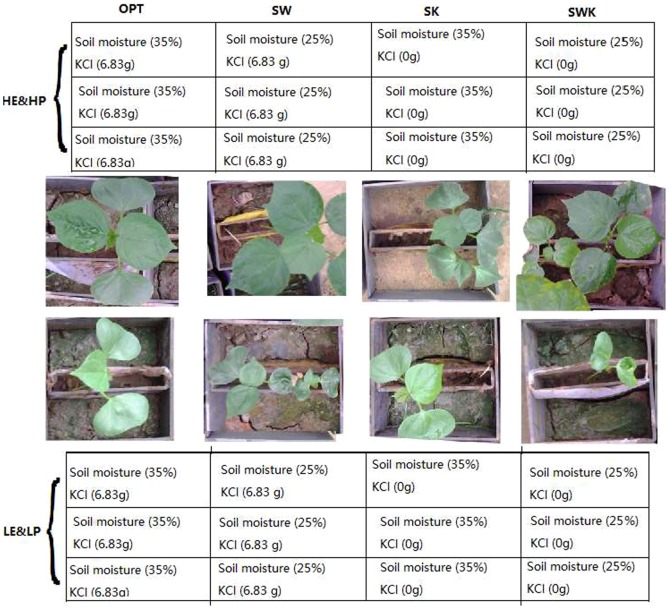
Diagram of experimental design to evaluate status changes of soil potassium.

For purpose of this study, we proposed a detail research scheme to evaluate status changes of soil potassium from path model (Fig. 1). We conducted soil K ions exchange experiments for soil samples, and further calculated their selectivity coefficients, and explored potassium status on basis of path model to determine dynamic changes of potassium status and specific environmental parameters. Soil samples were **N**on-**R**hizosphere soil in **OPT**imum of **K** of **H**igh efficiency genotype cotton (NROPTH); **N**on-**R**hizosphere soil in **OPT**imum of **K** of **L**ow efficiency genotype cotton (NROPTL); **N**on-**R**hizosphere soil in **S**hortage of **K** of **H**igh efficiency genotype cotton (NRSKH); **N**on-**R**hizosphere soil in **S**hortage of **K** of **L**ow efficiency genotype cotton (NRSKL); **N**on-**R**hizosphere soil in **S**hortage of **W**ater of **H**igh efficiency genotype cotton (NRSWH); **N**on-**R**hizosphere soil in **S**hortage of **W**ater of **L**ow efficiency genotype cotton (NRSWL); **N**on-**R**hizosphere soil in **S**hortage of **W**ater and **K** of **H**igh efficiency genotype cotton (NRSWKH); **Non-R**hizosphere soil in **S**hortage of **W**ater and **K** of **L**ow efficiency genotype cotton (NRSWKL); **R**hizosphere soil in **OPT**imum of **K** of **H**igh efficiency genotype cotton (ROPTH); **R**hizosphere soil in **OPT**imum of **K** of **L**ow efficiency genotype cotton (ROPTL); **R**hizosphere soil in **S**hortage of **K** of **H**igh efficiency genotype cotton (RSKH); **R**hizosphere soil in **S**hortage of **K** of **L**ow efficiency genotype cotton (RSKL); **R**hizosphere soil in **S**hortage of **W**ater of **H**igh efficiency genotype cotton (RSWH); **R**hizosphere soil in **S**hortage of **W**ater of **L**ow efficiency genotype cotton (RSWL); **R**hizosphere soil in **S**hortage of **W**ater and **K** of **H**igh efficiency genotype cotton (RSWKH); **R**hizosphere soil in **S**hortage of **W**ater and **K** of **L**ow efficiency genotype cotton (RSWKL). Firstly, we divided experiment soil into 8 treatments, and then implemented 256 soil samples test (Fig. 2). We measured 33 parameters of the different attributes of 256 soil samples. Those parameters were selected based on standards of Hashimoto and Kang research methods of phosphorus[19], [31]. Humic acid (NHA) in soil which can be extracted by NaOH solution (0.1 mol·L^−1^, pH = 3), humic acid (PHA) in soil which can be extracted by Na_2_P_2_O_4_ solution (0.05 mol·L^−1^, pH = 9.2), humus linked to iron (HMi) and humic linked to clay (HMc). Key experimental sampling test datasets were listed in Table 2–Table 6. Secondly, statistical analysis was further implemented to investigate relationship between environmental parameters and potassium status. Datasets were normalized, followed by correlation analysis (Table 7), 8 out of 16 parameters were selected thus to determine the most important parameters. Path coefficients of absolute value of represented the size effect on potassium morphology change. The size of the “+” meant the same as the arrow direction with arrow, “−” represents in contrast to the arrow direction with arrow. Finally, path model of potassium status was constructed on basis of direct and indirect correlated parameters. In this model, “e1” represents unknown variable and its impact factor, the straight line with arrows stands for direct impact factor, and double arc arrow is direction of interaction between parameters.

**Table 2:** Total contents of soil elements of Laboratory measurement for different parameters of 256 soil samples (R, rhizosphere soil; NR, non-rhizosphere).

Sample	Na%	Mg%	Al%	Si%	Fe%	Mg%	Ca%	Pppm	CO_3_%	Sppm	Clppm
**NR1-16**	0.44	0.39	6.49	36.86	3.35	1.49	0.37	491.01	49.74	219.34	119.8
**R17-32**	0.45	0.37	6.53	37.16	3.34	1.49	0.39	507.57	49.38	243.65	228.2
**NR33-48**	0.44	0.39	6.5	36.92	3.33	1.49	0.38	494.36	49.72	218.56	122.07
**R49-64**	0.44	0.39	6.51	36.94	3.34	1.49	0.39	483.04	49.64	251.93	217.78
**N65-80**	0.44	0.39	6.46	36.93	3.32	1.47	0.36	488.97	49.77	220.97	68.15
**R81-96**	0.47	0.42	6.3	36.38	3.29	1.46	0.4	515.89	50.45	248.77	80.32
**NR97-112**	0.44	0.38	6.49	37.04	3.35	1.48	0.37	506.34	49.62	218.38	82.48
**R113-128**	0.44	0.38	6.53	37.09	3.35	1.48	0.4	518.35	49.48	243.94	89.5
**NR129-144**	0.44	0.39	6.38	36.84	3.29	1.48	0.36	481.3	49.95	224.13	152.4
**R145-160**	0.45	0.39	6.39	36.94	3.27	1.47	0.38	512.73	49.84	237.7	201.2
**NR161-176**	0.43	0.38	6.49	37.09	3.32	1.49	0.37	484.59	49.58	225.39	142.88
**R177-192**	0.45	0.38	6.51	37.01	3.33	1.48	0.39	496.2	49.57	236.79	189.7
**NR193-208**	0.44	0.39	6.43	36.92	3.31	1.47	0.36	502.75	49.83	223.08	71.6
**R209-224**	0.45	0.38	6.43	37.08	3.32	1.46	0.39	491.22	49.64	245.74	81.48
**NR225-240**	0.44	0.38	6.46	37	3.31	1.47	0.38	509.11	49.7	228.9	90.5
**R241-256**	0.44	0.37	6.46	37.28	3.34	1.47	0.39	498.72	49.4	239.9	87.4

**Table 3:** Different forms of potassium of Laboratory measurement for different parameters of 256 soil samples (R, rhizosphere soil; NR, non-rhizosphere).

Sample	K(g/Kg)	wsK(ug/g)	neK(ug/g)	eK(ug/g)	wsK(ug/g, (weathered)	neK(ug/g) (weathered)	eK(ug/g) weathered
**NR1-16**	14.93	27.1	134.9	58.64	67.57	174.48	62.9
**R17-32**	14.9	12.03	54.21	14.32	59.36	155.15	66.59
**NR33-48**	14.9	20.01	134.13	50.84	76.97	177.27	55.87
**R49-64**	14.87	9.22	58.27	12.21	123.72	170.67	71.24
**N65-80**	14.7	24.8	121.55	40.27	53.02	190.22	59.93
**R81-96**	14.57	5.65	39.17	6.28	61.31	153.27	57.35
**NR97-112**	14.75	41.28	101.36	27.46	56.24	167.15	69.44
**R113-128**	14.8	6.05	42.83	5.23	58.41	149.91	52.24
**NR129-144**	14.77	38.55	158.29	56.41	68.09	190.55	65.03
**R145-160**	14.73	15.92	78.62	23.18	60.89	167.13	52.79
**NR161-176**	14.87	88.78	162.24	61.11	70.46	181.02	55.25
**R177-192**	14.83	18.61	70.07	19.27	64.82	185.07	59.89
**NR193-208**	14.73	13.26	99.74	30.21	63.65	171.35	62.95
**R209-224**	14.63	6.41	38.74	7.63	61.96	162.21	52.09
**NR225-240**	14.73	25.27	105.8	29.54	64.59	182.24	59.77
**R241-256**	14.73	6.56	44.8	5.31	72.96	154.08	60.7

**Table 4:** Silicate dissolving potassium bacteria of Laboratory measurement for different parameters of 256 soil samples (R, rhizosphere soil; NR, non-rhizosphere).

Sample	Bacteria of seedling stage (×10^4^ CFUg^−1^)	Bacteria of budding stage (×10^4^ CFUg^−1^)	Bacteria of wadding stage (×10^4^ CFUg^−1^)
**NR1-16**	0.8	2.1	1.6
**R17-32**	1.5	5.2	1.4
**NR33-48**	0.7	4.3	1.7
**R49-64**	1.1	3.4	1.9
**N65-80**	0.9	17	14
**R81-96**	1.0	87	30
**NR97-112**	1.2	26	19
**R113-128**	1.9	39	24
**NR129-144**	0.1	1.2	0.5
**R145-160**	0.4	1.3	0.7
**NR161-176**	0.9	4.3	2.2
**R177-192**	0.3	1.7	0.6
**NR193-208**	0.8	1.9	1.6
**R209-224**	0.7	7.1	4.0
**NR225-240**	0.1	2.3	0.9
**R241-256**	1.2	6.3	2.8

**Table 5:** Soil organic matter, humic acid, Total Carbon (TC), Total nitrogen (TN) of Laboratory measurement for different parameters of 256 soil samples (R, rhizosphere soil; NR, non-rhizosphere).

Sample	TC%	TN%	SOM(%)	PHA%	NHA%	Hmi%	HMc%
**NR1-16**	4.51E-03	1.71E-03	7.88	4.53	2.59	1.47	0.92
**R17-32**	4.41E-03	5.74E-03	5.67	4.84	3.52	0.33	0.33
**NR33-48**	5.34E-03	2.02E-03	7.32	3.48	3.67	0.42	0.37
**R49-64**	3.74E-03	1.04E-02	5.54	4.11	2.55	0.45	0.32
**N65-80**	4.50E-03	1.64E-03	8.01	5.33	3.89	1.35	1.24
**R81-96**	3.64E-03	4.24E-03	5.79	5.26	4.58	0.42	0.23
**NR97-112**	3.01E-03	3.93E-03	8.53	3.62	3.28	0.66	0.41
**R113-128**	2.80E-03	1.31E-02	5.70	4.41	3.68	0.46	0.27
**NR129-144**	3.52E-03	3.46E-03	8.30	4.46	3.70	0.35	0.28
**R145-160**	3.22E-03	2.02E-02	5.38	5.00	3.82	0.30	0.30
**NR161-176**	3.18E-03	3.97E-03	7.84	4.90	1.75	0.88	0.45
**R177-192**	3.12E-03	2.31E-02	6.79	6.89	3.95	0.34	0.26
**NR193-208**	3.57E-03	3.24E-03	8.45	4.57	3.82	0.25	0.19
**R209-224**	3.79E-03	2.67E-02	5.79	5.43	4.22	0.44	0.36
**NR225-240**	4.30E-03	3.83E-03	8.01	4.94	4.61	0.52	0.32
**R241-256**	4.37E-03	2.75E-02	6.23	4.70	4.08	0.36	0.18

**Table 6:** Weathering indices of soil minerals of Laboratory measurement for different parameters of 256 soil samples (R, rhizosphere soil; NR, non-rhizosphere).

Sample	ORP(eV)	pH	ba	Na/K	ICA	saf	CIA
**NR1-16**	17.30	7.27	0.45	0.29	1.03	8.73	76.08
**R17-32**	18.65	6.84	0.44	0.30	1.03	8.77	75.82
**NR33-48**	13.80	7.20	0.45	0.29	1.03	8.75	76.02
**R49-64**	30.24	6.5	0.45	0.30	1.04	8.74	75.95
**N65-80**	22.66	6.79	0.45	0.30	1.03	8.8	76.18
**R81-96**	22.68	6.73	0.48	0.32	1.08	8.86	75.08
**NR97-112**	10.22	6.94	0.44	0.30	1.03	8.78	76.21
**R113-128**	65.80	6.09	0.45	0.30	1.03	8.74	76
**NR129-144**	18.50	6.93	0.45	0.30	1.04	8.88	75.88
**R145-160**	80.84	5.79	0.46	0.31	1.04	8.90	75.67
**NR161-176**	31.46	6.48	0.44	0.29	1.02	8.80	76.16
**R177-192**	62.82	5.85	0.45	0.30	1.03	8.75	75.92
**NR193-208**	19.33	6.87	0.45	0.30	1.03	8.83	76.06
**R209-224**	52.48	6.19	0.45	0.31	1.04	8.86	75.76
**NR225-240**	13.72	6.98	0.45	0.30	1.03	8.83	75.99
**R241-256**	20.54	6.86	0.45	0.30	1.04	8.87	75.84

**Table 7:** Correlation analysis of environmental parameters of soil potassium.

	Z(SOM)	Z(CIA)	Z(Na/K)	Z(TC)	Z(TN)	Z(wsK)	Z(neK)	Z(eK)	Z(bacteria)	Z(ORP)	Z(pH)	Z(PHA)	Z(HMi)
**Z(CIA)**	0.61a												
**Z(Na/K)**	−0.52a	0.829b											
**Z(TN)**	−0.61a												
**Z(wsK)**	0.58a		−0.52a										
**Z(neK)**	0.80b	0.56a	−0.66b		−0.64b	0.77b							
**Z(ek)**	0.74b	0.51a	−0.67b		−0.62a	0.73b	0.98b						
**Z(bacteria)**	0.55a						0.53a						
**Z(ORP)**	−0.59a			−0.56a	0.66b								
**Z(pH)**	0.58a			0.66b	−0.68b					−0.95b			
**Z(NHA%)**											−0.57a		
**Z(PHA%)**		−0.51a	0.64			−0.65b		−0.51a					
**Z(HMi%)**								0.54a					
**Z(HMc%)**													0.93b
**Z(CO_3_)**		−0.62b	0.56						−0.50a				
**Z(K)**		0.53a	−0.79b									−0.77b	

(Note: statistically, probabilities of a and b: a, p<0.01; b, p<0.05)

## Results

### Path analysis of water-soluble potassium (wsK)

**Figure 3:**
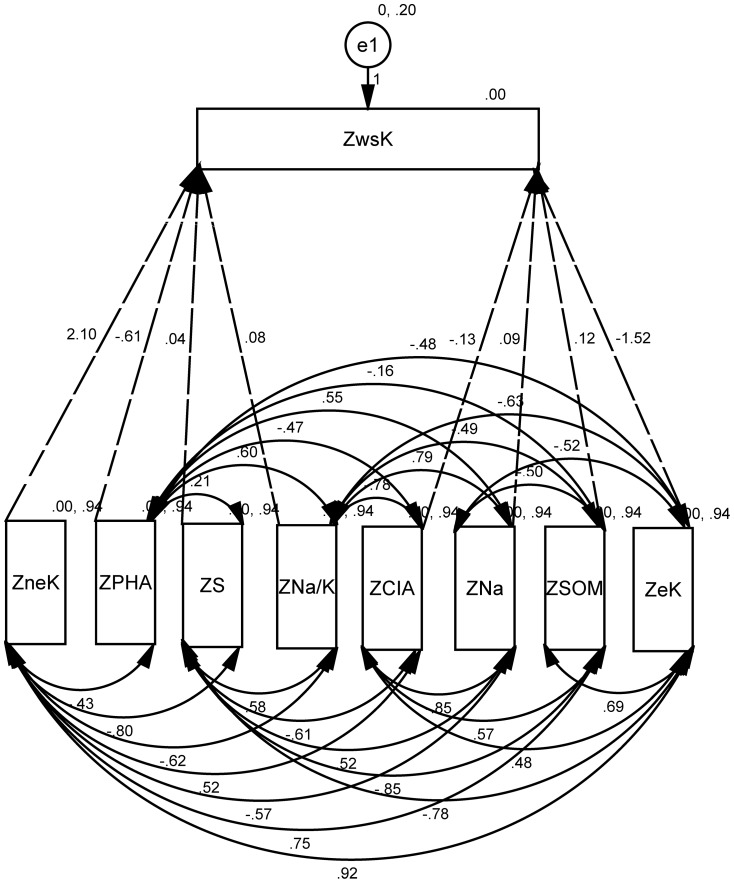
Path model of water-soluble potassium (wsK, solid line for double arrow, dashed-line for single arrow, and single arrow flows into ZwsK).

**Figure 4:**
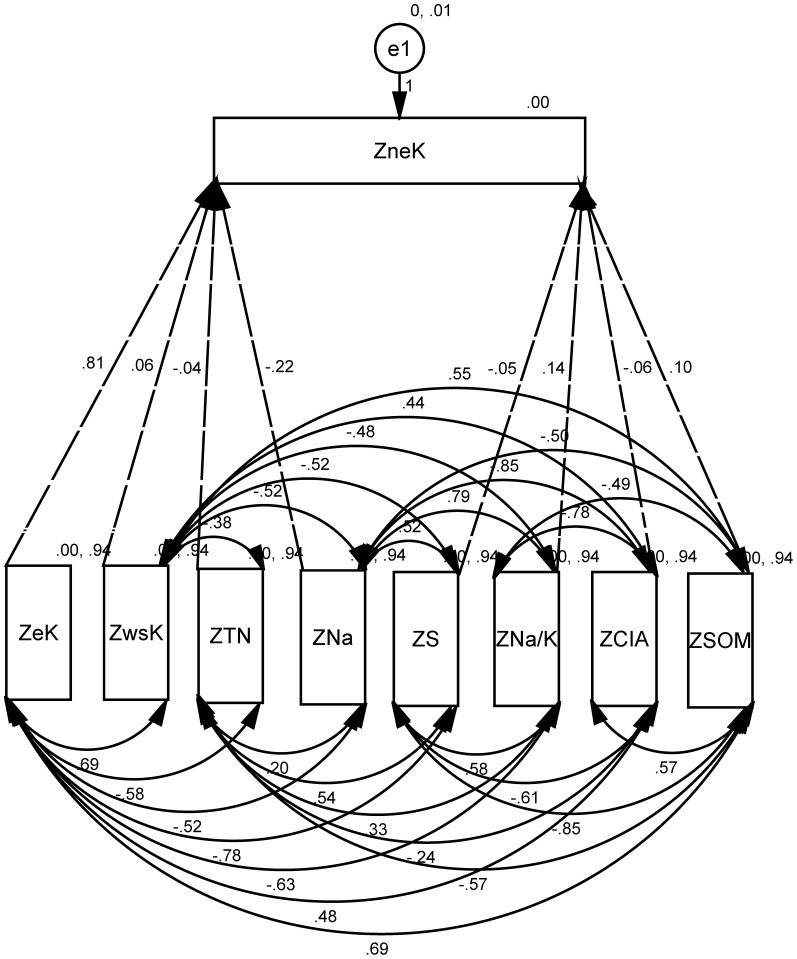
Path model of non-exchangeable potassium (neK, solid line for double arrow, dashed-line for single arrow, and single arrow flows into ZwsK).

**Figure 5:**
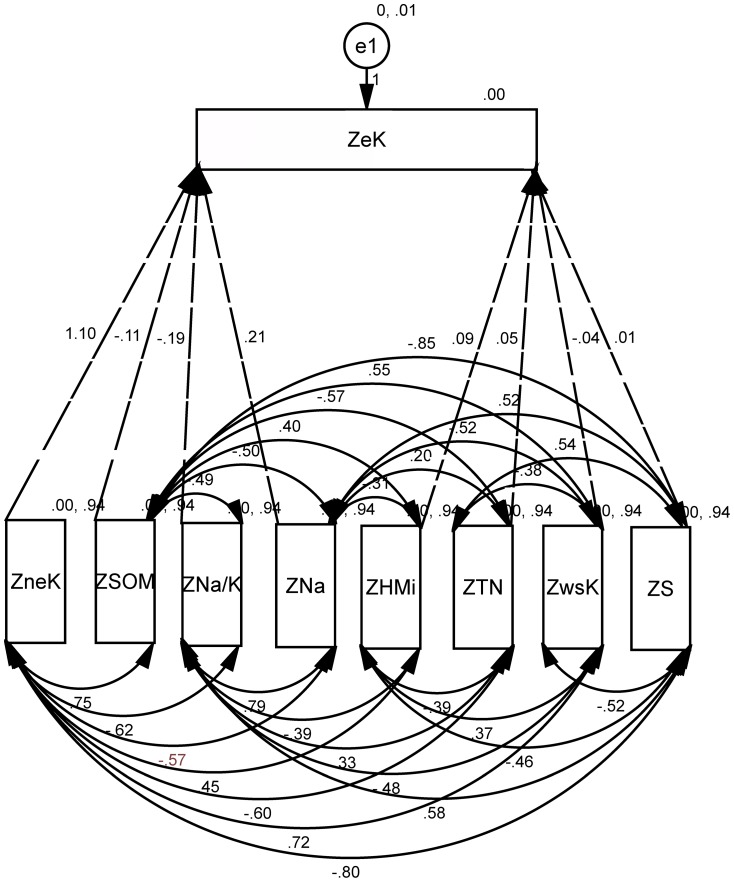
Path model of exchangeable potassium (eK, solid line for double arrow, dashed-line for single arrow, and single arrow flows into ZwsK).

For purpose of path analysis of water-soluble potassium, direct and indirect impact factors of the path analysis were derived from multiple linear regressions coefficients of wsK, and correlation coefficients between soil properties. Direct coefficient variables of wsK include neK, eK, PHA, SOM, S, Na and Na/K; while indirect coefficient variables of wsK include CIA, Na, Mg, CO_3_ and pH. Impact factor of path size of water-soluble potassium was determined by pH, CIA, Oxidation Reduction Potential (ORP), S, Na (Fig. 3 and Table 8). In the path model of wsK, direct effects of soil properties on the normalized value of wsK (ZwsK) were represented by single-headed arrows, while coefficients between soil properties were represented by double-headed arrows. Direct and indirect effects were indicated by value and marked with “±”. The direct effects of soil properties on the ZwsK were termed path coefficients and were standardized partial regression coefficients for each of the soil properties in the multiple linear regression against the ZwsK. Indirect effects of soil properties on the ZwsK were determined from the product of the simple coefficient between soil properties and the path coefficient (i.e., one two-headed arrow and one single-headed arrow). The coefficient between the ZwsK and soil property was the sum of the entire path connecting two variables, as described by
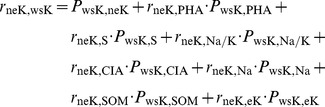
(1)where, *r_ij_* is the simple correlation between the ZwsK and a parameter of soil property, *P_ij_* is the path coefficient between the ZwsK and a parameter of soil property, and *r_ij_P_ij_* is the indirect effect of a parameter of on the ZwsK. An uncorrelated residue (*e_wsK_*) that represented the unexplained part of an observed variable in the path model was
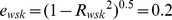
(2)where, *R_wsk_* ^2^ is the coefficient of determination of the multiple regression equation between the ZwsK and the eight soil properties. Those parameters of soil properties were neK, PHA, S, Na/K, CIA, Na, SOM, eK. Backward regression analysis was performed with the ZwsK as the dependent variable and the eight soil properties were regarded as independent variables. Backward regression was a multiple regression procedure in which all the independent variables were entered into the regression equation at the beginning, and variables that did not contribute significantly to the fit of regression model were successively eliminated until only statistically significant variables remain. Fig. 4 and Fig. 5 were similar expressions.

**Table 8:** Direct and indirect correlation coefficients of water-soluble potassium (*wsK*).

Variables	Direct correlation coefficients	Indirect correlation coefficients	Path contrition coefficients
**Z(PHA)**	−0.65	−0.32	−0.38
**Z(S)**	−0.56	0.17	−0.8
**Z(Na)**	−0.55	−1.15	−0.78
**Z(Na/K)**	−0.52	−0.01	−0.01
**Z(TN)**	−0.41	0.23	−0.38
**Z(ORP)**	−0.22	0.02	−1.28
**Z(Mg)**	−0.14	−0.79	−0.37
**Z(CO_3_)**	−0.07	−0.53	−0.24
**Z(pH)**	0.14	−0.35	−1.87
**Z(HMi)**	0.39	0.2	0.29
**Z(K)**	0.41	0.02	−0.01
**Z(CIA)**	0.47	2.02	−1.61
**Z(SOM)**	0.58	0.06	0.25
**Z(eK)**	0.73	0.05	0.14
**Z(neK)**	0.77		

In the path of water-soluble potassium, path contribution coefficients were quite different. Coefficient values were 2.10, −1.52 and 0.20 for wsK to neK, neK to wsK, and other variables to wsK respectively. It disclosed that most part of wsK was changed into neK (with values of 2.10). In the path of neK to wsK, coefficient was negative (with value of −1.52); it indicated that there was an inverse change from wsK to neK, a lot of wsK was changed into neK. Those dynamic changes indicated that wsK and eK reached equilibrium in soil solution, and this equilibrium represented its quick reaction process.

These changes impacted greatly on nutrients dynamic equilibrium of rhizosphere soil. This changed nutrients recycling process of the soil environment since continuous-release of humic acid had direct effects on absorption and uptakes of those variables including neK, eK and PHA, SOM, S, Na and Na/K. It was clear that rhizosphere effects were likely to result in changes in root exudates, pH and ORP (eV). Consequently, it led to changes in pH values and population, quality and activity of microorganisms while pH change was caused by root exuded organic acids. Under conditions of different pH values, humic acid significantly correlated to adsorption and desorption of potassium. Humic acid of potassium adsorption and desorption presented an upward trend when it increased in initial concentration (pH was 4.0–8.0), however, desorption rate declined. The migration distance also showed very significant linear relationship within ranges of migrations between water-soluble potassium and exchangeable potassium. Organic matter nutrients and chemical efficiency of humic acid altered different variables such as physical, chemical, and biotic in root-soil interface.

### Path analysis of non-exchangeable potassium (neK)

For path analysis of non-exchangeable potassium, we further investigated its direct and indirect impact factors. Results showed that direct coefficient variables of neK include eK, wsK, SOM, S, Na, Na/K, TN, CIA; and indirect coefficient variables of neK include Mg, CO_3_. Impact factor of path size of neK was determined by eK, CO3, pH (Fig. 4 Table 9). Table 9 showed there was significant correlation between eK and neK (with coefficient of 0.81). In the Path of non-exchangeable potassium, contribution coefficient was 0.01 for wsK to neK, although wsK was determined by general effects of N, Na, S and CIA. The SOM was also an important contributor to eK and wsK of rhizosphere soil. Coefficients were 0.69 and 0.55 between SOM and eK, SOM and wsK, respectively.

**Table 9:** Direct and indirect correlation coefficients of non-exchangeable potassium (*neK*).

Variables	Direct correlation coefficients	Indirect correlation coefficients	Path contrition coefficient
**Z(S)**	−0.86	0.19	−0.25
**Z(Na/K)**	−0.66	0.02	0.03
**Z(TN)**	−0.64	0.03	−0.03
**Z(Na)**	−0.61	−0.34	−0.37
**Z(PHA)**	−0.46	0.03	0.16
**Z(ORP)**	−0.41	0.10	−0.42
**Z(Mg)**	0.04	−0.67	−0.34
**Z(CO3)**	0.10	1.18	0.56
**Z(K)**	0.44	−0.14	0.2
**Z(pH)**	0.46	−0.16	−0.50
**Z(HMi)**	0.48	0.07	0.06
**Z(CIA)**	0.56	0.05	−0.07
**Z(wsK)**	0.77	0.01	0.01
**Z(SOM)**	0.8	−0.11	−0.14
**Z(eK)**	0.98	0.76	0.69

The correlation between the ZneK and a soil property was the sum of the entire path connecting two variables, as described by
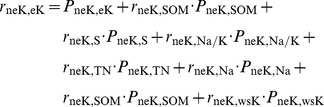
(3)where, *r_ij_* is the simple correlation coefficient between the ZneK and a soil property, *P_ij_* is the path coefficient between the ZneK and a soil property, and *r_ij_P_ij_* is the indirect effect of a soil property on the ZneK. An uncorrelated residue (*e*) that represents the unexplained part of an observed variable in the path model was
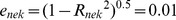
(4)where *R_nek_* ^2^ is the coefficient of determination for the multiple regression equation between the ZneK and those soil properties. Those soil properties were wsK, SOM, S, Na/K, TN, Na, SOM, eK.

It disclosed that plant nutrition was indirectly influenced by nutrients release of microbial decomposition and organic matter. It was particularly obvious in nutrition release of low molecular weight organics in rhizosphere soil. High contents of associated ions (Na^+^) were limit factors that control crop yields and soil nutrients use. Ammonium nitrogen was likely to reduce fixation of potassium ions of soil, and increase risk of potassium leaching. NH_4_^+^ and K^+^ were competitive ions for absorption of ORP (eV) of eK and neK in rhizophere soil, and different status of nitrogen effects on efficient use of potassium. Therefore, suitable proportion of N and K is important for plant. Soil nitrogen availability determines potassium absorption of root surface and cell membrane. Root membrane could directly affected by NH_4_^+^, NO_3_^−^, K^+^ when it absorbs potassium ions, and indirect affected by potential balance from assimilation of NH_4_^+^. Strong affinity of K^+^, high efficiency and fast speed of absorption of potassium are likely to improve efficient absorption of potassium.

### Path analysis of exchangeable potassium (eK)

As to exchangeable potassium, it was quite different situation in comparison with those of water soluble and non-exchangeable potassium. It showed that direct coefficient variables of eK include neK, wsK, SOM, S, Na, Na/K, TN, CIA, HMi, PHA; and indirect coefficient variables of eK include Mg, CO_3_. Impact factor of path size of eK was determined by neK, CO_3_, pH, ORP, Na (Table 10).

**Table 10:** Direct and indirect correlation coefficients in exchangable potassium (*eK*).

Variables	Direct correlation coefficients	Indirect correlation coefficients	Path contrition coefficient
**Z(S)**	−0.83	−0.28	0.36
**Z(Na/K)**	−0.67	−0.03	−0.04
**Z(TN)**	−0.62	−0.04	0.04
**Z(Na)**	−0.55	0.46	0.54
**Z(PHA)**	−0.51	−0.06	−0.24
**Z(ORP)**	−0.38	−0.16	0.6
**Z(Mg)**	0.07	0.95	0.49
**Z(CO3)**	0.11	−1.7	−0.81
**Z(pH)**	0.47	0.23	0.71
**Z(K)**	0.48	0.21	−0.3
**Z(CIA)**	0.51	−0.07	0.1
**Z(HMi)**	0.54	−0.09	−0.09
**Z(wsK)**	0.73	−0.01	−0.02
**Z(SOM)**	0.74	0.18	0.2
**Z(neK)**	0.98	1.49	1.44

From Fig. 5, it showed that neK was closely related to wsK (with correlation coefficients of 0.72), and there was an inverse proportion between ZneK and ZwsK (the normalized value of neK and wsK) in condition of changes of micro-bioorganic and geochemical environmental factors in rhizosphere soil. Path contribution coefficients were 1.10 and 0.04 for neK to eK, and wsK to eK. It suggests that neK is more likely to be changed into eK while the possibility of wsK to eK is relatively low.

The correlation between the normalized value of eK (ZeK) and a soil property is the sum of the entire path connecting two variables, as described by
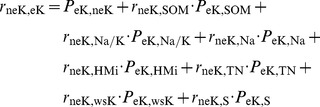
(5)where *r_ij_* is the simple correlation coefficient between the ZeK and a soil property, *P_ij_* is the path coefficient between the ZeK and a soil property, and *r_ij_P_ij_* is the indirect effect of a soil property on the ZeK. An uncorrelated residue (*e*) that represents the unexplained part of an observed variable in the path model was
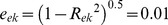
(6)Where, *R_eK_* ^2^ is the coefficient of determination of the multiple regression equation between the ZwsK and the eight soil properties. Those soil properties are neK, SOM, Na/K, Na, HMi, TN, wsK, S. Backward regression analysis was performed with the ZeK as the dependent variable and the eight soil properties as independent variables. Backward regression is a multiple regression procedure in which all the independent variables are entered into the regression equation at the beginning, and variables that do not contribute significantly to the fit of the regression model are successively eliminated until only statistically significant variables remain.

Cation exchange capacity (CEC) or the number of exchangeable cation depends on the type and number of clay and organic matter in the soil. To a certain extent, TN, SOM, HMi and the PHA and the CIA are able to well reflect type and number of clay and organic matte in rhizosphere soil. Proper pH value of solution, hot water pressure, concentration increases of magnesium carbonate and ion are useful to exchangeable potassium (neK) when it is absorbed by crops. A plenty of CO_2_ could be produced by root respiration and soil microbe respiration, and is fixed in form of CO_3_^2−^. For stable pH values, it is beneficial for organic acid to reach in equilibrium of absorption in soil solution. The exchangeable potassium and fixed potassium which comes from layers of secondary clay mineral are released when there occurs hydrolysis and cation substitution resulted by chemical element H^+^. Those variables, such soil organic matter (ZSOM), iron-binding Humic (ZHMi), and water-soluble nitrogen (ZTN), not only provide nutrition for cotton root, but also changed soil chemical compositions and absorption characteristics of potassium in rhizosphere soil. Additionally, concomitant phenomenon (ZS) appears between S (the lost chemical element in soil) and potassium. Antagonism occurred between Na and K, and it implied that sodium salt was able to have significant effect on plant (ZNa/K, ZNa).

From above analysis, we conclude that key direct factors of wsK, neK, and eK were S, Na/K, the CIA, SOM, Na, and TN, and key indirect factors were CO_3_, Mg, pH, Na, S, and SOM. Whereas, direct impact factors of water soluble potassium (wsK) were PHA, S, Na, Na/K, K, the CIA, SOM, eK and neK. Each of its impact value was −0.65, −0.56, −0.55, −0.52, 0.41, 0.47, 0.58, 0.73, and 0.77 respectively. Indirect impact factors were Na, Mg, CO_3_, pH, PHA, S, HMi, TN and CIA. Each of its impact value was −1.15, −0.79, −0.53, −0.35, −0.32, 0.17, 0.2, 0.23, and 2.02, respectively. Non exchangeable potassium direct impact factors were: S, Na/K, Na, TN, the CIA, wsK, SOM and eK. Each of its impact value was −0.86, −0.66, −0.64, −0.61, 0.56, 0.77, 0.8, and 0.98 respectively. Indirect impact factors were CO_3_, eK, S, SOM, K, pH, Na and Mg. Each of its impact value was 1.18, 0.76, 0.19, −0.11, −0.14, −0.16, −0.34, and −0.67 respectively. Exchangeable potassium direct impact factors were S, Na/K, Na, TN, HMi, wsK, SOM and neK. Each of its impact value was −0.83, −0.67, −0.62, −0.55, 0.54, 0.73, 0.74, and 0.98 respectively. Indirect impact factors were CO_3_, S, SOM, K, pH, Na, Mg and neK. Each of its impact value was −1.7, −0.28, 0.18, 0.21, 0.23, 0.46, 0.95, and 1.49 respectively. Absolute values of path coefficients represent the change ability of the potassium morphology, and the bigger value means the stronger ability to change potassium morphology. And “+”with and the arrow direction represent change direction of potassium morphology. In this way, we can quantitatively describe the K elements morphological changes.

### Path model of soil potassium status changes of cotton

It is of great importance to further investigate on the equilibrium movement in soil. The following part will discuss the shift of dynamic balance among wsK, eK and neK, and their flow path. To test the reliability of the models, we collect another 48 cotton soil samples in the same experiment, using stepwise regression method, observation of potassium mobility and morphological change.

**Figure 6:**
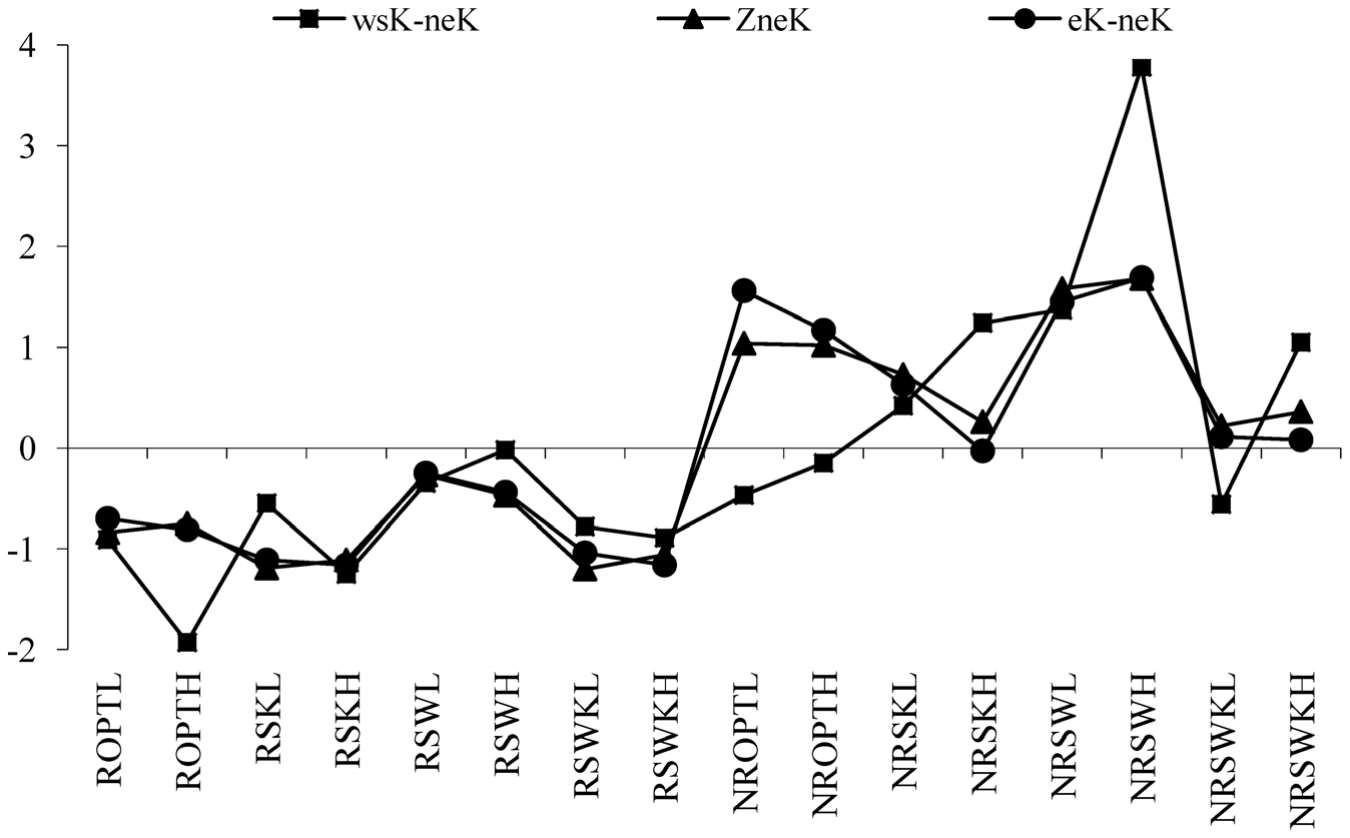
Dynamic equilibrium of non-exchangeable potassium in rhizosphere/non-rhizosphere soils.

We carried out a plot experiment of potash application with high K-efficiency genotype (HEG) and low K-efficiency genotype (LEG) cotton. The soil in each treatment weights 8.5 kg. Detail fertilizer schemes are listed in Table 11. LEG and HEG cottons' planting effects is shown in Fig. 6.

**Table 11:** Fertilizing rates of different treatments (unit: g).

Treatments	OPT	SW	SK	SWK
**Urea**	6.65	6.65	6.65	6.65
**Na_2_PO_4_.2H_2_O**	11.72	11.72	11.72	11.72
**KCl**	6.83	6.83	0	0
**CaCO_3_**	2.13	2.13	2.13	2.13
**H_3_BO_3_**	1.00	1.00	1.00	1.00
**ZnSO_4_.7H2O**	1.87	1.87	1.87	1.87
**MgSO_4_.7H2O**	2.61	2.61	2.61	2.61

*OPT*, optimum fertilization treatment; *SW*, water limited; *SK*, potassium limited; *SWK*, water & potassium limited.

(1) Equilibrium shift in the path for non-exchangeable potassium

Supposed that V_wsK_→_nek_ is path for wsK to neK, V_ek_→_nek_ is path for eK to neK, therefore, equilibrium shift in path for non-exchangeable potassium can be expressed by contribution coefficient functions as below,
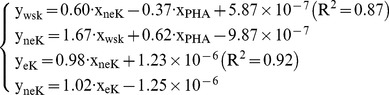
(7)


Equilibrium occurs when X_wsK_, X_PHA_, X_eK_ satisfy with Function 8,
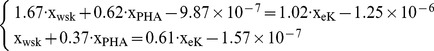
(8)


Here, y_nek_ is the equilibrium threshold. And the value of x_wsK_, x_PHA_, x_eK_ is determined by soil characteristics. Calculated results from Equation 7 were listed in Table 12. In the table, column 1 and 2 were calculated from Equations 7. We used the wsK, eK, neK to construct dynamic equilibrium equations (
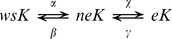
).The column 3 was normalized values of neK and the column 4 was the direction of balance movement of neK. These three column datasets were presented in Fig. 6.

**Table 12:** Dynamic equilibrium of exchangeable/non-exchangeable potassium (*neK/eK*) in rhizosphere/non-rhizosphere soils.

Variables	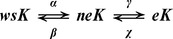			Movement direction at balance state	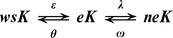			Movement direction at balance state
	V_wsk→neK_	V_ek→neK_	ZneK		V_wsk→eK_	V_nek→eK_	ZeK	
**RSWKH**	−0.89	−1.16	−1.06	β,χ	−0.78	−1.39	−1.14	θ, ω
**RSWKL**	−0.78	−1.04	−1.2	α, χ	−0.78	−1.51	−1.02	θ, λ
**RSWH**	−0.02	−0.44	−0.47	α, χ	−0.19	−0.5	−0.44	θ, λ
**RSWL**	−0.35	−0.25	−0.27	α, χ	−0.32	−0.28	−0.24	θ, λ
**RSKH**	−1.25	−1.16	−1.11	β, χ	−0.8	−1.79	−1.14	θ, ω
**RSKL**	−0.55	−1.11	−1.19	β, γ	−0.82	−1.19	−1.09	θ, λ
**ROPTH**	−1.93	−0.81	−0.75	β, χ	−0.65	−2.11	−0.79	θ, ω
**ROPTL**	−0.91	−0.70	−0.84	β, γ	−0.51	−1.48	−0.69	θ, λ
**NRSWKH**	1.05	0.08	0.36	β, χ	0.14	1.43	0.08	ε, λ
**NRSWKL**	−0.56	0.11	0.22	α, χ	−0.45	0.54	0.11	θ, ω
**NRSWH**	3.78	1.69	1.68	β, χ	3.24	1.28	1.66	ε, λ
**NRSWL**	1.37	1.45	1.58	α, χ	0.79	2.73	1.42	θ, λ
**NRSKH**	1.24	−0.03	0.26	β, χ	0.92	0.17	−0.03	ε, λ
**NRSKL**	0.42	0.63	0.73	β, χ	0.11	1.45	0.61	θ, ω
**NROPTH**	−0.15	1.17	1.02	β, γ	−0.12	1.76	1.14	θ, ω
**NROPTL**	−0.47	1.56	1.04	β, γ	0.23	0.90	1.53	θ, ω

In the reversible equilibrium of 
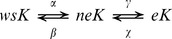
, soil samples RSWKL, RSWH, RSWL, NRSWKL, NRSWL, K balance cycle were moved in α, χ direction movement; soil samples RSWKH, RSKH, RSKL, ROPTH, ROPTL, NRSWKH, NRSWH, NROPTH, NROPTL, K balance cycle were moved in β and γ direction movement; soil samples NRSKH, NRSKL, K balance cycle were moved in β, χ direction movement.

In Fig. 6, neK reaches its equilibrium in following soil types which includes LEG in water stress treatment (SW), HEG after potassium stress treatment (SK), LEG after optimum fertilization treatment (OPT), and LEG after water stress treatment (SW). However, all the value in those soils with HEG in SW, LEG in SK, HEG in SW and SK, the values were all greater than the equilibrium, wsK reached its saturation. For other treatment soils, when wsK was lower than the equilibrium and PHA and eK was greater than equilibrium, neK changed into wsK. It proves that eK equales to total K in matured crops. When eK is below a certain level in soil, plants can no longer get eK. When eK was at low levels, it was absorbed by other ions with stronger absorption. This reduces chance of potassium to enter into the soil solution. In this condition, neK begins to release through low concentrations of wsK and eK. However, the release capacity of neK depends on situations of crop types

(2) Equilibrium shift in the path for exchangeable potassium

Supposed that V_wsK_→_ek_ is path for wsK to eK, V_nek_→_ek_ is path for neK to eK, therefore, equilibrium shift in path for exchangeable potassium can be expressed by contribution coefficient functions as below,
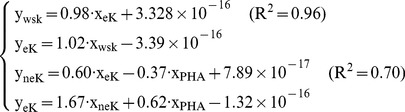
(9)


**Figure 7:**
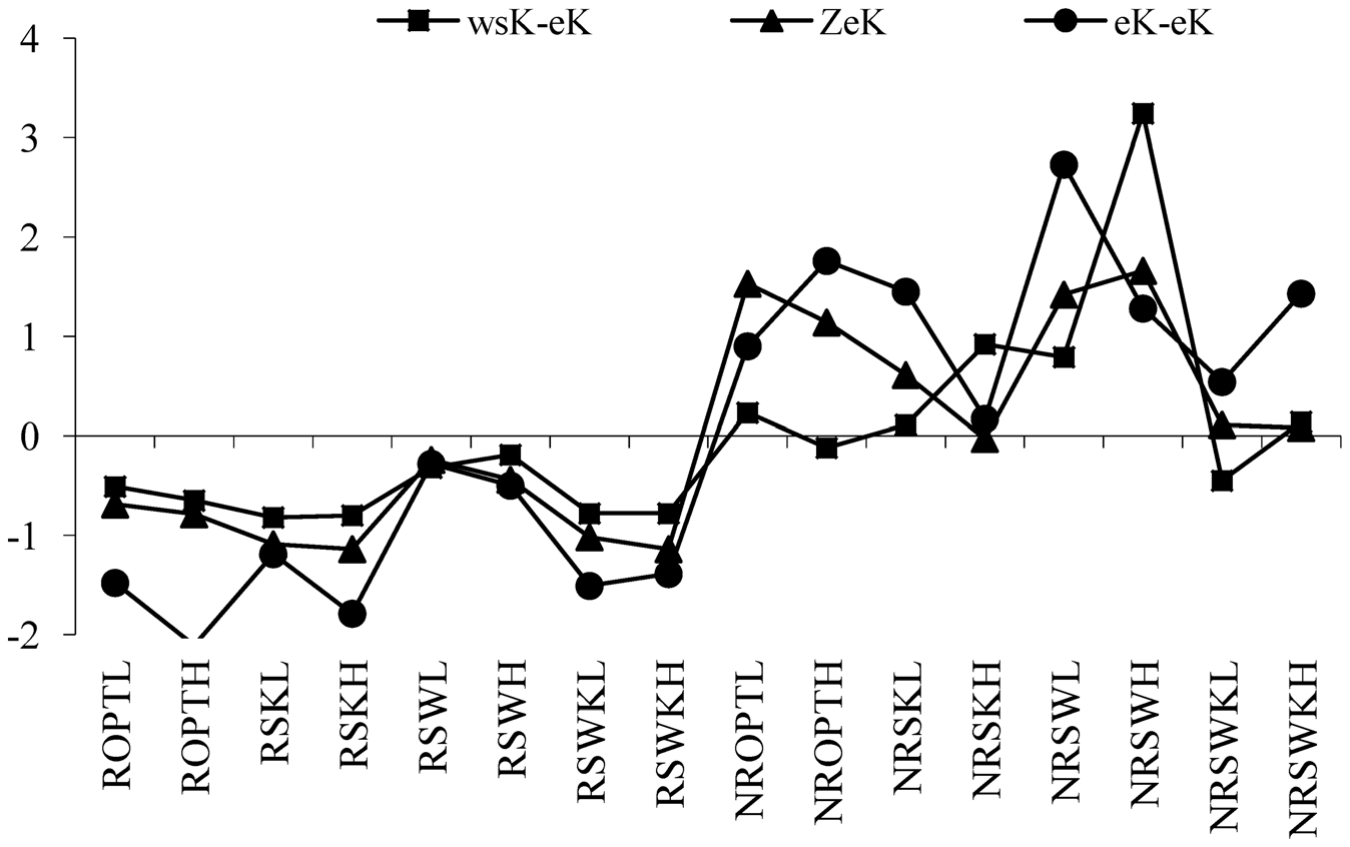
Dynamic equilibrium of exchangeable potassium in rhizosphere/non-rhizosphere soils.

Equilibrium occurs when x_wsK_, x_PHA_, x_neK_ satisfy with Functions 11,
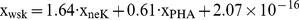
(10)Here, y_ek_ is the equilibrium threshold. And the value of x_wsK_, x_PHA_, x_neK_ is determined by soil characteristics. Calculated results from Equations 9 and 10 are listed in Table 12. In this table, column 5 and 6 are calculated from Equations 9 and 10. We use the wsK, eK, neK constructs the dynamic equilibrium equations (
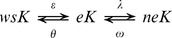
).The column 7 is normalized values of neK and the column 8 is the direction of movement of balance of eK. These three column datasets are described in Fig. 7.

In the reversible equilibrium of 
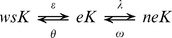
, soil samples SWH, NRSWL, K balance cycle were moved in ε, λ direction movement; soil samples RSWKL, RSWH, RSWL, K balance cycle were moved in θ, λ direction movement; soil samples RSWKH RSKH, RSKL ROPTH, ROPTL, NRSWKH, NRSKH, NRSKL, NROPTH, NROPTL K balance in soil samples, K balance cycle were moved in θ, ω direction movement respectively.

As is shown in Fig. 7, neK reaches its equilibrium in following soil plots, including LEG (SW), HEG (SK), LEG (OPT), and LEG (SW). And the exchangeable K reaches its equilibrium in soil types of LEG (SW). For other treatment soils, such as LEG (SW and SK), LEG (SW), LEG (SK), HEG (SW and SK), HEG (OPT), neK changes to eK more or less. In soil of HEG (SW and SK), eK changes to wsK when wsK was lower than equilibrium. In soil of LEG (SW and SK), neK changes to eK when wsK of soil was greater than that of crops. Soils of both genotypes cotton (HEG and LEG) can efficiently absorb exchangeable potassium.

During a lot of vermiculite in the soil, those negative charges caused by vermiculite isomorphous substitution are near p-site, the electrostatic attraction of potassium ion became bigger, the adsorption capacity much more than other type (2∶1) minerals, therefore, wsK and neK are fixed and the soil changed into deficiency. In general, drying accelerated the fixation of wsK adsorption, but neK still moved in the direction of eK in the reversible equilibrium in HEG rhizosphere soil because rhizosphere soil exchangeable potassium content was low to meet the needs of the cotton growth in the SW and SWK plant. When moisture was adequate, redox reaction of soil became strengthened and oxidation reduction potential was reduced. For example, Fe^2+^ and Mn^2+^ ions were increased rapidly, Fe^2+^ and Mn^2+^ replaced p-site potassium of soil colloids and part of i-site potassium, and this improved the content of rapidly-available potassium and biological effective parts. In addition, the dissolved iron and manganese mineral alteration and also increased the release of mineral K.

## Discussion and Conclusions

In this study, changes of potassium status in soil were investigated through our proposed path model on basis of laboratory experimental datasets. Through this investigation, following findings were achieved.

Firstly, changes of potassium status in the rhizosphere soil were controlled by different environmental variables. wsK, neK and eK were controlled by some common factors and have their speculiar characteristics respectively. Those parameters, such as soil organic matter (SOM), Na and Na/K and S, were significant direct coefficient factors which control dynamic process of wsK, neK and eK. CO_3_ and Mg are significant indirect coefficient factors which control dynamic of wsK, neK and eK. The pH is the primary factor which controls path of the wsK, neK and eK. Organic acids of HEG root system secretion increased soil acidity; the amount of fixed potassium was reduced. PHA surface is porous structure, PHA increased the adsorption of wsK in the experiment, and HMi could promote the moving to wsK, eK in the K form exchange. Both of them were negative correlation significantly negative correlation. First, under acid condition, hydroxyl aluminum and aluminum ions can occupy the potassium fixation point stronger. At the same time, the larger diameter of hydroxy aluminium ions entered into the interlayer of mineral, formed as the “island” and played a supporting role, provided the potassium ion diffusion of mobile channel in the interlayer. These reduced the soil potassium fixation. Second, water protonated in acidic condition, the radius of H_3_O^+^ 12–13.3 nm were similar to K^+^, H_3_O^+^ can produced competitive adsorption in soil where fixed on K^+^ parts, so potassium reduced fixation. Those dynamic processes, such as eK to wsK, low concentration wsK and eK to neK, are likely to occur, in condition that wsK is lower than equilibrium value and PHA and eK are greater than equilibrium values. eK is changed neK when wsK is greater than equilibrium value. In soil of HEG (SW and SK), eK is changed into wsK when plant wsK is lower than soil wsK. In soil of LEG (SW and SK), neK is changed into eK when plant wsK is higher than soil wsK. Both genotype cottons can effectively absorb eK in treatment of stress of potassium (SK).

Secondly, it discloses that SOM, S, TN, HMi and PHA in rhizosphere soil determined dynamic balance of potassium status which includes adsorption and desorption, precipitation and dissolution, complexion and chelating. Special characteristics of humus, e.g., acidic and hydrophilic, cationic exchange, complexing capacity and high absorption capacity, could improves the sustained-release effect of potassium fertilizer. Many variables, pH, ORP, etc., are closely related to transformation and utilization of potassium fertilizer. The pH change in soil is mainly caused by coupling effects of nutrient uptake by plant roots and the secretion of organic acid and absorption imbalance of positive ion and negative ion leads to rhizosphere pH change, however, different factors affect imbalance of absorption of positive ion and negative ion. Dynamic equilibrium of potassium is affected by equilibrium constants, temperature, and products of this equilibrium system (wsK). Therefore, it is useful for cotton planting to improve to appropriate temperature, to alter SOM, TN and pH in soil, and to reduce the equilibrium constant to positive reaction. Meanwhile, it is good to choose HEG cotton for better use soil wsK. It is important to understand ways to slow down the fast response and accelerate speed of slow response in potassium balance system.

Finally, path model can effectively determine direction and quantities of potassium status changes between eK, neK and wsK under influences of specific environmental parameters. The proposed path model was able to quantitatively disclose moving direction of K status and quantify its equilibrium threshold. It provided a theoretical and practical basis for scientific and effective fertilization in agricultural plants growth. The significance of this study is that we are able to implement investigation on the equilibrium movement of potassium status through path model analysis. It discloses the gradual dynamic process of potassium status, and to decouple the sophisticated interactions between different variables. It is useful to guide the use of potassium fertilizer for cotton crops in practical. The model provides a biogeochemical theory basis of controlling moisture in cotton plant, applying potash fertilizer and organic fertilizer, and appropriately reducing nitrogen fertilizer.

However, environmental parameters such as soil moisture and temperature are some of the most important factors that influenced crop growth. Observation of dynamic change of soil moisture and temperature in every other time-step is quite sophisticated for us to design the control experiments while it involves in more than 4 times treatments, which means that we have more than 500 pots. By limitation, we had to set the soil moisture into two states, water sufficient (>35%) and deficient (<25%), and temperature were set to indoor temperature. Anyway, we were very aware of the importance of soil moisture and temperature impacts on control experiment potassium status. The next step of our work is to further investigate efficient utilization of potassium and the influence of soil moisture and temperature on exchange of potassium forms. We hope this study is able to gain knowledge of promoting high efficient use of potassium for crops.

## Supporting Information

Appendix S1 **Explanation of symbols.**
(DOC)
