# Correction: Evaluating Status Change of Soil Potassium from Path Model

**DOI:** 10.1371/annotation/4e8e4899-e014-4b98-962d-d2bf44a35229

**Published:** 2013-12-13

**Authors:** Wenming He, Fang Chen

There are errors in the legends of Figures 4 and 5, in both cases the last word is incorrect.

The correct legend of Figure 4 is: Path model of non-exchangeable potassium (neK, solid line for double arrow, dashed-line for single arrow, and single arrow flows into ZneK).

The correct legend of Figure 5 is: Path model of exchangeable potassium (eK, solid line for double arrow, dashed-line for single arrow, and single arrow flows into ZeK). 

