# Supplementary material for: Evaluating Status Change of Soil Potassium from Path Model
Source: PLoS One. 2013 Oct 30;8(10):e76712. doi: 10.1371/journal.pone.0076712 (PMC3813672; doi:10.1371/journal.pone.0076712)
Supplement: Appendix S1 — Explanation of symbols. (DOC) [file pone.0076712.s001.doc]

**Explanations of symbols**

CIA, chemical index of alteration,

CO_3_: Carbonate,

eK, Exchangeable K (potassium), neK, Non-exchangeable K (potassium), wsK, water-soluble potassium,

humus linked to iron (HMi), humic linked to clay (HMc).,

HEG, High Efficiency Genotype, LEG, Low Efficiency Genotype,

Na/K, ratio of sodium-potassium,

NROPTH, **N**on-**R**hizosphere soil in **OPT**imum of **K** of **H**igh efficiency genotype cotton,

NROPTL, **N**on-**R**hizosphere soil in **OPT**imum of **K** of **L**ow efficiency genotype cotton,

NRSKH, **N**on-**R**hizosphere soil in **S**hortage of **K** of **H**igh efficiency genotype cotton,

NRSKH, **N**on-**R**hizosphere soil in **S**hortage of **K** of **H**igh efficiency genotype cotton,

NRSKL, **N**on-**R**hizosphere soil in **S**hortage of **K** of **L**ow efficiency genotype cotton,

NRSKL, **N**on-**R**hizosphere soil in **S**hortage of **K** of **L**ow efficiency genotype cotton,

NRSWKH, **N**on-**R**hizosphere soil in **S**hortage of **W**ater and **K** of **H**igh efficiency genotype cotton,

NRSWKL, **Non-R**hizosphere soil in **S**hortage of **W**ater and **K** of **L**ow efficiency genotype cotton,

ORP, Oxidation Reduction Potential,

PHA, humic acid from sodium pyrophosphate,

ROPTH, **R**hizosphere soil in **OPT**imum of **K** of **H**igh efficiency genotype cotton,

ROPTL, **R**hizosphere soil in **OPT**imum of **K** of **L**ow efficiency genotype cotton,

RSKH, **R**hizosphere soil in **S**hortage of **K** of **H**igh efficiency genotype cotton,

RSKL, **R**hizosphere soil in **S**hortage of **K** of **L**ow efficiency genotype cotton,

RSWH, **R**hizosphere soil in **S**hortage of **W**ater of **H**igh efficiency genotype cotton,

RSWKH, **R**hizosphere soil in **S**hortage of **W**ater and **K** of **H**igh efficiency genotype cotton,

RSWKL, **N**on-**R**hizosphere soil in **S**hortage of **W**ater and **K** of **L**ow efficiency genotype cotton,

RSWKL, **R**hizosphere soil in **S**hortage of **W**ater and **K** of **L**ow efficiency genotype cotton,

RSWL, **R**hizosphere soil in **S**hortage of **W**ater of **L**ow efficiency genotype cotton,

SWH, **S**hortage of **W**ater of **L**ow efficiency genotype cotton,

SWL, **S**hortage of **W**ater of **L**ow efficiency genotype cotton,

SOM, Soil Organic Matter in soil solution,

TN, total nitrogen in soil solution,

ZwsK, ZeK, ZneK, the normalized value of wsK, eK and neK.
